# Graft orientation using the over‐the‐top technique in anterior cruciate ligament reconstruction: Magnetic resonance imaging‐based study

**DOI:** 10.1002/jeo2.70744

**Published:** 2026-05-06

**Authors:** Ying Ren Mok, Alberto Grassi, Giulio Maria Marcheggiani Muccioli, Andrea Pierangeli, Lidia Ana Martín Domínguez, Claudio Rossi, Stefano Zaffagnini

**Affiliations:** ^1^ Department of Orthopaedic Surgery National University Hospital Singapore Singapore; ^2^ Clinica Ortopedica e Traumatologica II, IRCCS Istituto Ortopedico Rizzoli Bologna Italy; ^3^ Dipartimento di Scienze Biomediche e Neuromotorie (DIBINEM) University of Bologna Bologna Italy; ^4^ Institut Català de Traumatologia i Medicina de l'Esport (ICATME) Hospital Universitari Quirón‐Dexeus, Universitat Autònoma de Barcelona Barcelona Spain

**Keywords:** anatomic reconstruction, coronal inclination angle, graft inclination angle, graft orientation, over‐the‐top technique, sagittal inclination angle

## Abstract

**Purpose:**

To quantify sagittal and coronal inclination angles following over‐the‐top (OTT) anterior cruciate ligament (ACL) reconstruction using magnetic resonance imaging (MRI), to compare these measurements with the native ACL, and to evaluate graft alignment relative to the European Society of Sports Traumatology, Knee Surgery and Arthroscopy (ESSKA) consensus.

**Methods:**

Thirty‐five patients underwent single‐bundle OTT ACL reconstruction combined with lateral plasty and underwent postoperative MRI evaluation at a minimum of 6 months. The reference group consisted of 28 patients with knee pain and an intact ACL on MRI. Sagittal and coronal inclination angles were measured using standardised tibial‐axis–based MRI definitions. Measurements were performed independently by two examiners, and mean values were used for analysis. Group comparisons were conducted using independent‐samples *t*‐tests. Interexaminer reliability was assessed using intraclass correlation coefficients (ICC).

**Results:**

OTT reconstruction demonstrated a mean sagittal inclination angle (SIA) of 53.2° ± 5.1° and a mean coronal inclination angle (CIA) of 71.3° ± 5.9°. Compared with the native ACL cohort, OTT reconstructions showed significantly greater sagittal (mean difference 3.6°, *p* = 0.007) and coronal (mean difference 8.2°, *p* < 0.001) inclination angles. Most reconstructions fell within ESSKA consensus sagittal (<60°) and coronal (<75°) MRI thresholds (85.7% and 71.4%, respectively). Interexaminer agreement was excellent for both sagittal and coronal measurements (ICC > 0.90).

**Conclusion:**

OTT ACL reconstruction produces graft inclination angles that differ significantly from native ACL orientation but frequently fall within consensus‐recommended MRI thresholds for sagittal alignment. MRI‐based inclination assessment provides objective structural characterisation of this technique and complements previously reported long‐term clinical outcomes. However, these imaging findings do not establish full anatomic replication or clinical equivalence.

**Level of Evidence:**

Level IV, case series.

**Abbreviations**
CIA: coronal inclination angle
OTT: over‐the‐top
SIA: sagittal inclination angle

## INTRODUCTION

Anterior cruciate ligament (ACL) reconstruction is one of the most commonly performed procedures in sports medicine, with increasing incidence driven by participation in pivoting sports. Efforts to improve outcomes have focused on reproducing the functionally important fibres [24] of the native ACL—particularly the direct [37, 39] and indirect [23] fibres—through optimal femoral tunnel placement [11]. This has driven adoption of the concept of ‘anatomic’ ACL reconstruction [7], which remains variably defined and largely comparative [49].

Anatomic reconstruction encompasses femoral and tibial footprint coverage, three‐dimensional graft orientation and restoration of physiologic knee kinematics. In clinical practice, however, imaging‐based parameters are frequently used as reproducible surrogates of graft alignment. Since the early 1990s, magnetic resonance imaging (MRI)‐derived [13] sagittal and coronal inclination angles (SIA and CIA) have been reported for both native and reconstructed ACLs and correlate with femoral tunnel position on CT [20] and computer‐assisted navigation [52] data. Although these measures do not fully characterise graft function, they provide objective descriptors of orientation that allow standardised comparison.

Despite ongoing refinements in drilling techniques, femoral tunnel malposition remains the most common cause of technical failure in ACL reconstruction [44, 48]. Data from the MARS cohort [50] indicate that malposition accounts for approximately 80% of technical failures and necessitates creation of a new femoral tunnel in the majority of revision cases [36]. Transitions to anteromedial portal drilling have improved femoral tunnel placement and graft orientation compared with earlier transtibial techniques [30], as reported in multiple studies and systematic reviews. However, variability [21] in tunnel positioning persists [40], particularly during the learning phase [31] and malposition remains a recognised cause of graft failure. Furthermore, flexible and retrograde drilling systems may introduce additional technical complexity [26, 46] and cost considerations [10]. These ongoing challenges raise the question of whether approaches that eliminate the need for a femoral tunnel warrant reconsideration.

The Marcacci–Zaffagnini over‐the‐top (OTT) technique [34], used continuously at the Rizzoli Orthopaedic Institute since 1993, avoids femoral tunnel drilling [55]. Despite favourable clinical outcomes in adolescents, skeletally mature individuals [54] and elite athletes [6], the absence of a femoral tunnel has led to its frequent classification as ‘non‐anatomic’, and objective radiologic assessment of graft orientation has been limited. Furthermore, historical concerns regarding rotational control underscore the importance of clarifying graft alignment using reproducible imaging parameters.

Therefore, the purpose of this study was threefold: (1) to quantify the SIA and CIA following OTT ACL reconstruction using MRI‐based measurements; (2) to compare these measurements with those of the native ACL within the same cohort and (3) to interpret the observed graft orientation relative to established 2022 European Society of Sports Traumatology, Knee Surgery and Arthroscopy (ESSKA) consensus targets [47] and the range of orientations reported for contemporary ACL reconstruction techniques.

We hypothesised that Marcacci–Zaffagnini OTT ACL reconstruction would achieve SIA and CIA within the range of native ACL orientation and within consensus‐recommended thresholds for acceptable graft alignment on MRI.

## METHODS

### Patients and procedures

This case series represents a secondary analysis of patients from an institutional anterior cruciate ligament reconstruction cohort established at the Istituto Ortopedico Rizzoli to evaluate outcomes following ACL reconstruction, including OTT techniques. The study protocol was approved by the Area Vasta Emilia Centro Ethics Committee (PG 0000445), and written informed consent was obtained from all participants.

The study population comprised 35 consecutive patients who underwent OTT ACL reconstruction between April 2014 and July 2021, as well as 30 control participants. Controls consisted of patients who underwent knee MRI for non‐instability‐related symptoms and demonstrated an intact native ACL without prior ligament or meniscal surgery.

As the primary objective of this study was imaging‐based analysis, inclusion criteria for the surgical cohort included a minimum postoperative follow‐up of 6 months and consent to undergo postoperative MRI. Exclusion criteria included prior ACL or meniscal surgery, open physes and multiligament knee injuries. Patients from either group were excluded if MRI examinations were incomplete or did not allow reliable measurement of SIA and/or CIA.

### Surgical technique

All patients underwent the same surgical procedure, consisting of a single‐bundle OTT ACL reconstruction combined with lateral extra‐articular reconstruction using single‐strand gracilis and semitendinosus hamstring tendons as described previously [34].

### The tendons were harvested with preservation of their tibial attachments

**Figure 1:**
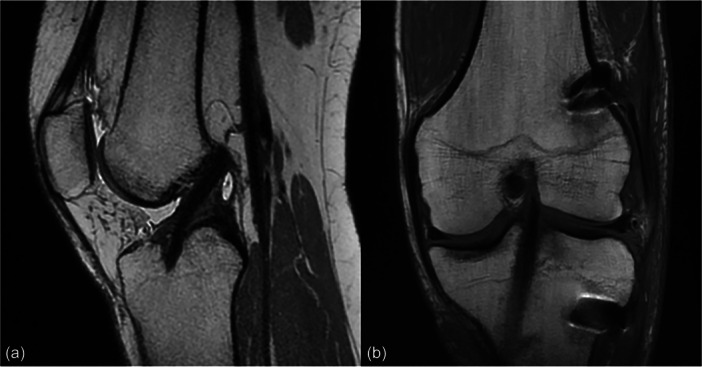
Postoperative MRI scan of a patient following over‐the‐top ACL reconstruction in sagittal view (a) and coronal view (b). ACL, anterior cruciate ligament; MRI, magnetic resonance imaging.

Graft diameter was determined intraoperatively according to tendon size and patient anatomy and ranged between 7 and 9 mm. The single‐strand grafts were passed through the tibial tunnel and routed over the top of the femur. With the knee positioned at 90° of flexion and 45° of external tibial rotation, a posterior drawer force was applied and the grafts were fixed under maximal manual tension to the femoral cortex using two bone staples. The remaining portion of the graft was then passed deep to the iliotibial band, superficial to the lateral collateral ligament, and fixed to the Gerdy tubercle with a single staple to complete the lateral extra‐articular reconstruction. Fixation was performed with the knee at 90° of flexion and neutral tibial rotation. Figure 1 shows a representative postoperative MRI scan of a patient following OTT ACL reconstruction. All procedures were performed by two senior knee surgeons experienced in the OTT technique, following a standardised institutional protocol.

### Radiologic measurements

**Figure 2:**
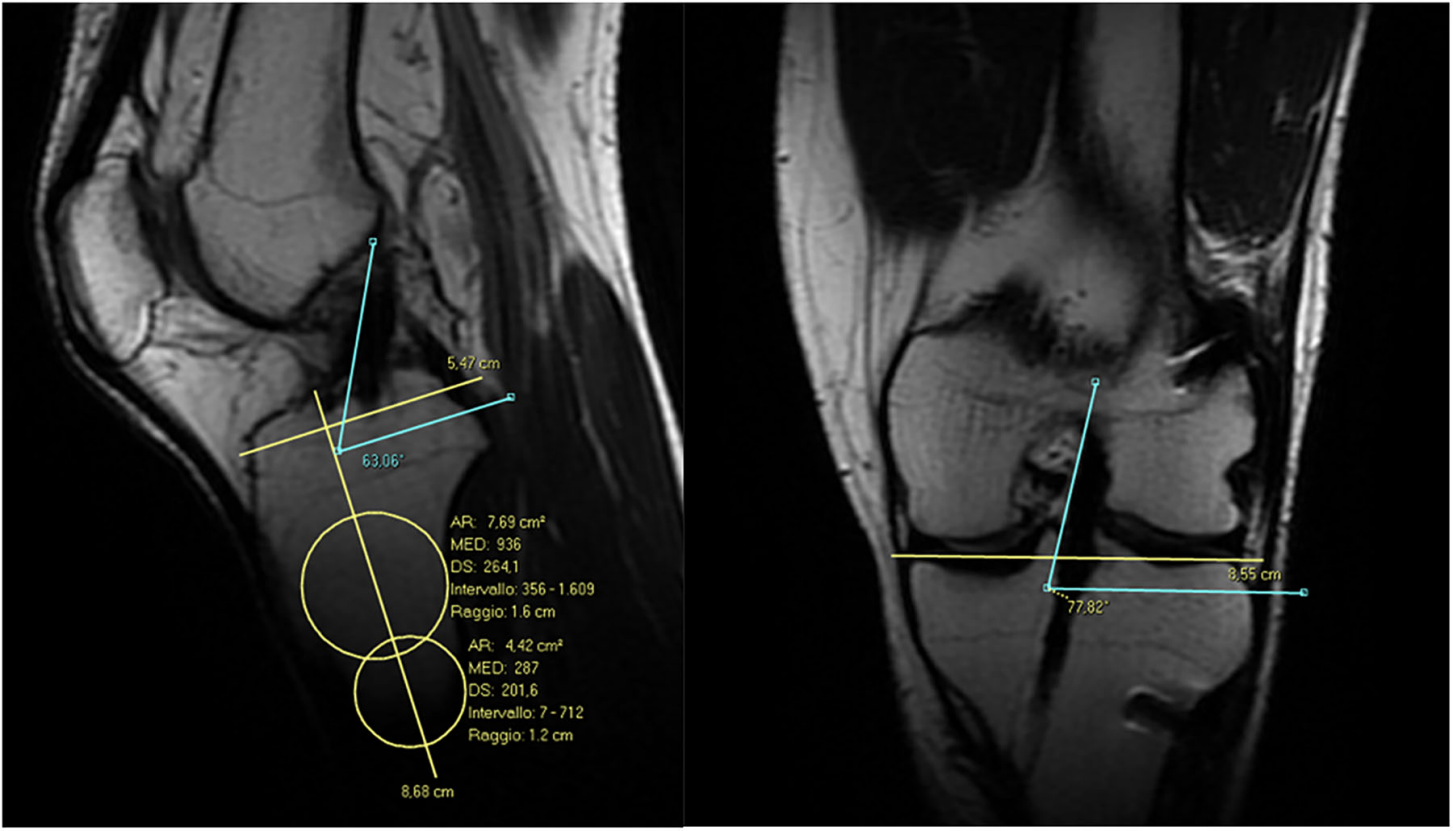
Tibia axis‐based measurement of sagittal inclination angle. Coronal inclination angle measured referencing from tibia plateau. AR, area, expressed in cm^2^; DS, standard deviation; MED, median pixel intensity.

ACL orientation was assessed on MRI in sagittal and coronal planes. SIA was defined as the angle between the anterior margin of the ACL (or graft) and a line perpendicular to the tibial long axis on the sagittal image best visualising the ligament, as described by Mellado et al [35]. The tibial axis was determined using a validated diaphyseal circular or linear method and aligns with ESSKA consensus recommendations [47] (Figure 2).

In the coronal plane, the entire ACL could rarely be visualised on a single slice. Therefore, the medial margin of the ligament was tracked across sequential coronal images. The medial edge of the femoral ACL attachment was first identified and marked, after which the images were scrolled anterior–posteriorly to locate the medial edge of the tibial ACL attachment. A line connecting these two points, representing the medial border of the ACL, was then compared with the articular surface of the tibial plateau to determine the CIA [41] (Figure 2).

### Comparison of native and reconstructed ACL graft inclination

All measurements were performed independently by two orthopaedic residents on two separate occasions, separated by a 1‐month interval. Examiners were blinded to group allocation during measurement. Slice selection followed a predefined protocol: sagittal measurements were performed on the slice best visualising the full longitudinal extent of the ligament, and coronal measurements were obtained by sequential tracking of the medial border of the ligament from femoral to tibial insertion. Knee position at the time of MRI acquisition followed institutional standard imaging protocols, with patients scanned in full extension. To reduce measurement variability, both observers underwent a calibration session prior to formal measurement. Measurement methodology was reviewed and confirmed by a senior knee surgeon with extensive experience in ACL imaging prior to final analysis.

Mean values from all measurements were calculated and used for comparative analysis between the native ACL and reconstructed graft groups.

### Comparison of reconstructed ACL graft inclination with ESSKA consensus

For consensus‐based comparison, the SIA and CIA were evaluated relative to the ESSKA‐consensus [47]. Grafts were considered to meet consensus‐recommended alignment if both observers independently recorded a SIA ≤ 60° and a CIA ≤ 75°; grafts exceeding either threshold were classified as outside recommended limits.

### Sample size calculation and statistical analysis

The secondary imaging analysis was not powered to detect small differences between groups but was designed to provide a descriptive evaluation of graft orientation relative to native ACL values and consensus imaging targets. Sample size selection was informed by prior work from Jamsher et al. [22], who demonstrated excellent measurement reliability and meaningful between‐group differences in SIA and CIA using cohorts of 18 patients per group. Accordingly, the present study's larger cohorts were considered sufficient to evaluate MRI‐based graft orientation and support comparative analysis. Statistical analyses were performed using MedCalc statistical software (version 22.023; MedCalc Software Ltd). Continuous variables are reported as mean ± standard deviation, and categorical variables as counts and percentages. Between‐group comparisons were conducted using independent‐samples *t‐*tests. To quantify the magnitude of differences between groups, effect sizes were calculated using Cohen's *d*. Ninety‐five per cent confidence intervals (95% CI) were computed for mean differences to estimate precision. Statistical significance was defined as *p* < 0.05.

## RESULTS

### Study population

A total of 35 patients were screened for inclusion in the OTT ACL reconstruction group, while 30 individuals were screened for the native ACL reference group. Two patients were excluded from the native ACL group due to motion artifacts that precluded reliable inclination angle measurement. The final cohorts, therefore, comprised 35 patients in the reconstruction group and 28 in the native ACL reference group. Demographic characteristics, including age (mean ± SD) and sex, are presented in Table 1. The reconstruction group was younger and had a higher proportion of male patients compared with the native ACL reference group. Given the exploratory imaging‐based objective of the study, no adjusted analysis was performed; therefore, between‐group comparisons should be interpreted as descriptive associations rather than causal technique effects.

**Table 1:** Demographic characteristics of patients.

Group	*N*	Age	Males	Females
Native ACL	28	33.1 ± 12.8	17	11
Over‐the‐top	35	26.1 ± 8.1	29	6

Abbreviation: ACL, anterior cruciate ligament.

### Reliability

MRI‐based measurements demonstrated excellent reproducibility, supporting internal consistency of angle quantification but not implying clinical equivalence. Interobserver agreement was excellent, with intraclass correlation coefficients (ICCs) of 0.94 for sagittal inclination angle and 0.93 for coronal inclination angle.

### Inclination angles of native ACL and following OTT reconstruction

In the reconstruction group, the mean sagittal graft inclination angle (mean of both observers) was 53.2° ± 5.1°, compared with 49.6° ± 5.6° in the native ACL group. The mean difference was 3.6° (95% CI, 0.86°–6.34°; *p* = 0.007), corresponding to a moderate effect size (Cohen's *d* = 0.68).

The mean coronal graft inclination angle was 71.3° ± 5.9° in the reconstruction group and 63.1° ± 5.5° in the native ACL group. The mean difference was 8.2° (95% CI, 5.32°–11.08°; *p* < 0.001), representing a large effect size (Cohen's *d* = 1.43).

These findings indicate that although inclination angles differed statistically between groups, the magnitude of difference was moderate in the sagittal plane and larger in the coronal plane. This is demonstrated in Table 2.

**Table 2:** Comparison of sagittal and coronal inclination angles between native ACLs and over‐the‐top ACL reconstructions.

Variable	Native ACL	Over‐the‐top	Mean difference (95% CI)	Cohen's *d*	*p*‐value
SIA (°)	49.6 ± 5.6	53.2 ± 5.1	3.6 (0.86–6.34)	0.68	0.007
CIA (°)	63.1 ± 5.5	71.3 ± 5.9	8.2 (5.32–11.08)	1.43	<0.001

*Note*: Values are presented as mean ± standard deviation. Mean differences are calculated as OTT minus Native ACL. Confidence intervals derived using Welch's *t*‐test. Effect size reported as Cohen's d.

Abbreviations: ACL, anterior cruciate ligament; CI, confidence interval; CIA, coronal inclination angle; SIA, sagittal inclination angle.

**Figure 3:**
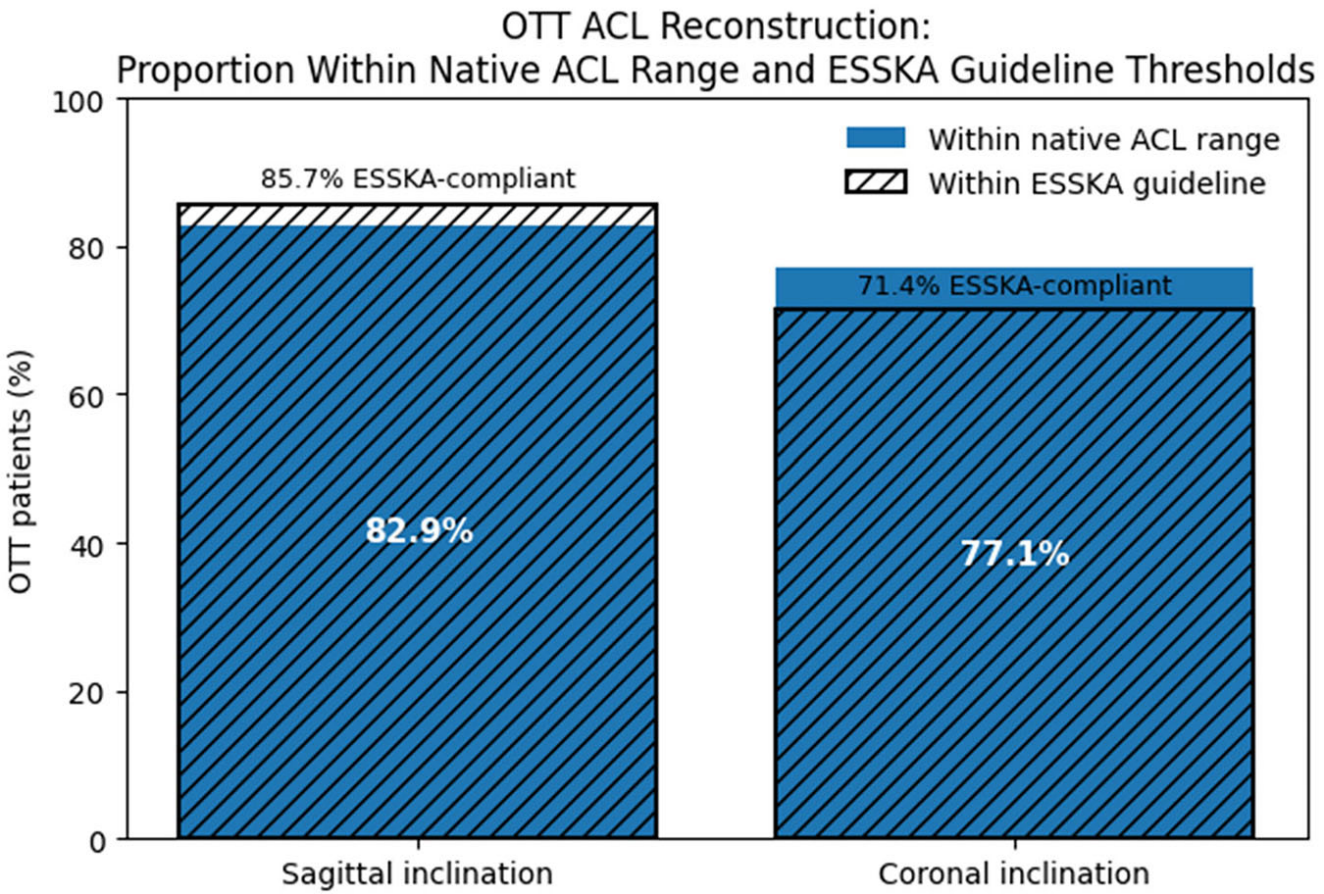
Evaluation of SIA and CIA against native ACL range and ESSKA. Proportion of over‐the‐top (OTT) ACL reconstructions with sagittal and coronal graft inclination angles falling within the native ACL range (solid bars) and within ESSKA consensus thresholds (hatched overlay). Native reference ranges were defined using the minimum and maximum values observed in the native ACL cohort based on the mean of two independent examiners. ACL, anterior cruciate ligament; CIA, coronal inclination angle; ESSKA, European Society of Sports Traumatology, Knee Surgery and Arthroscopy.

Using the observed minimum and maximum values within the native ACL cohort as an internal reference range, 82.9% (29 out of 35) of OTT reconstructions demonstrated SIA within the native range, while 77.1% (27 out of 35) demonstrated CIA within the native range. This approach provides a descriptive internal benchmark but should not be interpreted as a population normative interval. Based on ESSKA consensus thresholds, 30 out of 35 patients (85.7%) demonstrated a SIA ≤ 60° and 25 patients (71.4%) demonstrated a CIA ≤ 75° (Figure 3).

## DISCUSSION

This study demonstrated that SIA and CIA following Marcacci–Zaffagnini OTT ACL reconstruction were significantly greater than those observed in the native ACL group, with moderate and large effect sizes in the sagittal and coronal planes, respectively. Therefore, OTT graft orientation should not be interpreted as identical to native ACL anatomy. However, most reconstructions met ESSKA consensus MRI thresholds (<60° sagittal, <75° coronal), indicating that sagittal alignment, in particular, remained within ranges considered acceptable for graft positioning. Mean SIA and CIA were 53.2° ± 5.1° and 71.3° ± 5.9°, respectively, with 85.7% of reconstructions meeting sagittal and 71.4% meeting coronal guideline criteria.

The concept of ‘anatomic’ ACL reconstruction extends beyond inclination angles alone and may include femoral and tibial footprint coverage, bundle‐specific placement, three‐dimensional graft trajectory and functional behaviour under load. The present study assessed graft orientation using MRI‐based SIA and CIA as reproducible surrogate descriptors; it does not establish full anatomic replication nor functional equivalence.

The native ACL SIA observed in this study (49.6° ± 5.6°) aligns with previously reported [1, 4, 12, 16, 20, 22, 35, 41, 45] MRI‐based values obtained using similar measurement methodologies, although higher values [1] have been reported in some populations. The mean native CIA (63.1° ± 5.5°) was on the lower end among prior studies, which may partly explain why some OTT reconstructions fell outside the native coronal range. Variability in CIA likely reflects methodological differences, despite excellent interobserver agreement. The above studies are summarised in Supplementary Table S1.

While sagittal inclination has been associated in prior studies with femoral tunnel malposition and adverse outcomes, the present investigation did not evaluate clinical endpoints and therefore cannot infer clinical acceptability based solely on inclination measures. Higher SIA values on MRI have been associated with nonanatomic graft placement due to femoral tunnel malposition [20] and with adverse biomechanical [17] and clinical outcomes [18]. Conversely, excessively low SIA values have also been implicated, with Li et al. [27] demonstrating a strong negative association between SIA and graft signal–noise quotient at 7 months, suggesting impaired graft maturation following ACL reconstruction. In contrast, evidence linking CIA to clinical outcomes remains limited [12, 32]. It is therefore of interest to examine how OTT ACL reconstruction compares with other techniques with respect to these angles. Interobserver agreement was high in the present study, consistent with prior reports [16, 22, 26, 45], indicating that inclination angles are reproducibly measured on routine MRI and supporting comparison with previously published MRI‐based studies.

Although relatively few investigations have reported SIA and CIA across multiple femoral drilling techniques using standardised tibial‐axis–referenced methods, the characteristics of these studies [1, 2, 3, 4, 9, 12, 16, 20, 22, 26, 42, 45, 51] are summarised in Table S1. To reduce methodological heterogeneity, only studies employing similar MRI measurement frameworks were considered for contextual comparison. These comparisons are descriptive and do not represent a formal systematic review or meta‐analysis.

**Figure 4:**
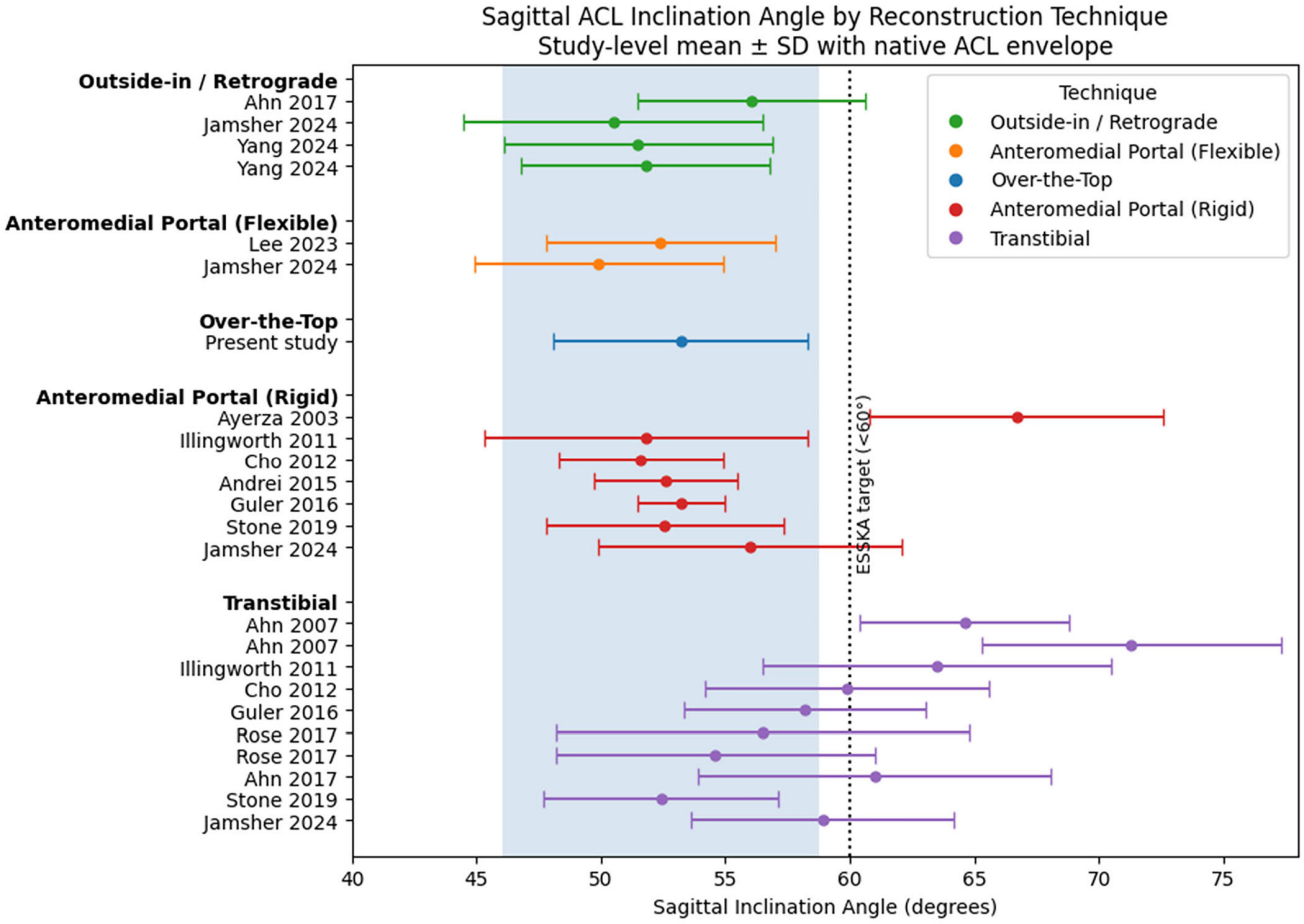
ACL Graft SIA by reconstruction technique. Study‐level mean ± standard deviation values are shown for different ACL reconstruction techniques. The shaded band represents the range of sagittal inclination angles reported for the native ACL, and the vertical dotted line indicates the ESSKA consensus (<60°). ACL, anterior cruciate ligament; CIA, coronal inclination angle; ESSKA, European Society of Sports Traumatology, Knee Surgery and Arthroscopy; SD, standard deviation.

When viewed alongside the cohort reported by Jamsher et al. [22]—which evaluated outside‐in, anteromedial portal (flexible and rigid) and transtibial techniques—the SIA observed in the present study falls within the range reported for outside‐in and anteromedial portal approaches. In contrast, higher SIA values were reported for transtibial drilling in several cohorts. Figure 4 illustrates study‐level mean ± standard deviation values for each technique relative to native ACL ranges and ESSKA consensus thresholds. These visual comparisons are intended for contextual interpretation rather than controlled head‐to‐head inference.

**Figure 5:**
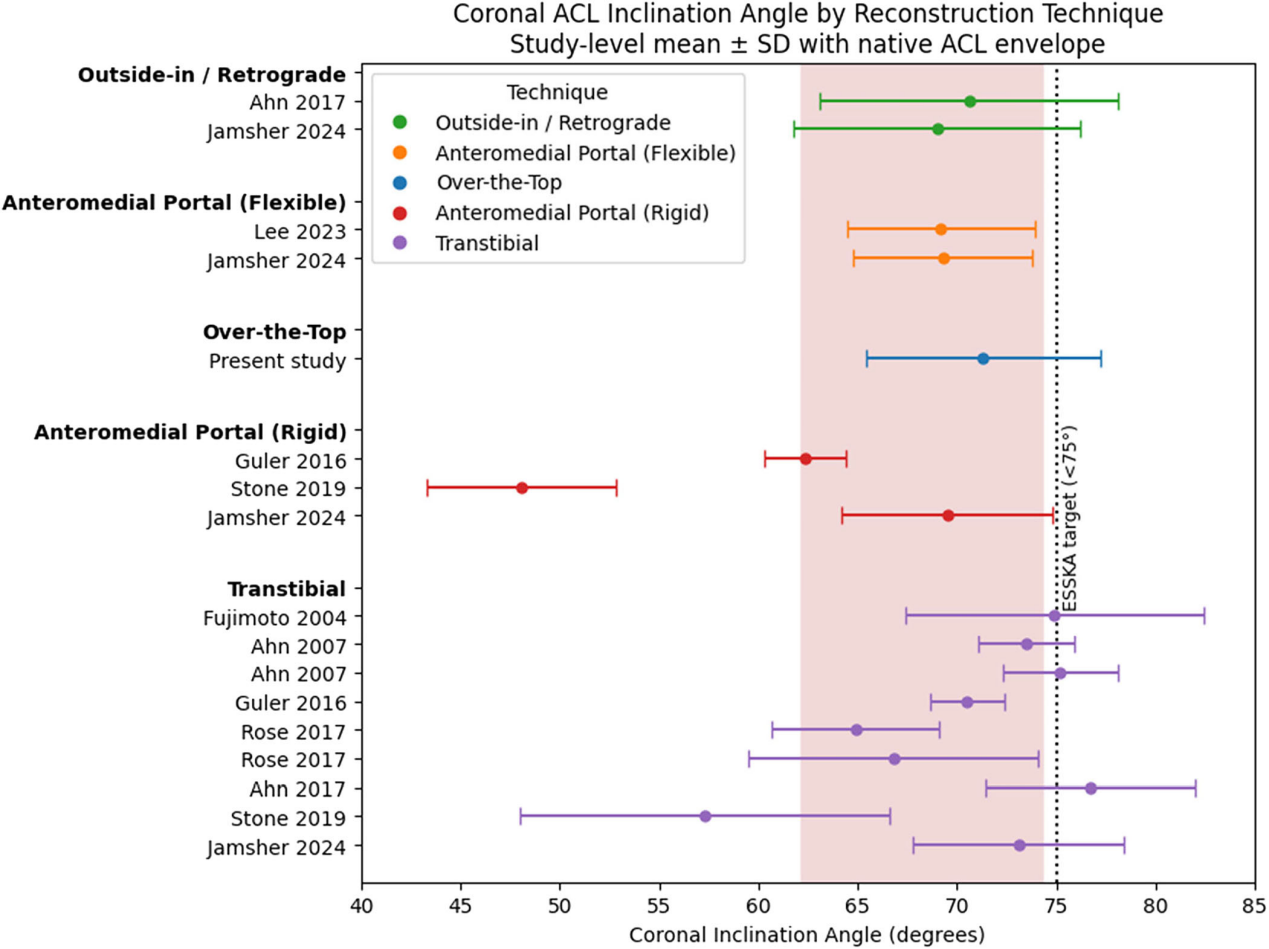
ACL Graft CIA by reconstruction technique. Study‐level mean ± standard deviation values are shown for different ACL reconstruction techniques. The shaded band represents the range of coronal inclination angles reported for the native ACL, and the vertical dotted line indicates the ESSKA consensus threshold (<75°). ACL, anterior cruciate ligament; ESSKA, European Society of Sports Traumatology, Knee Surgery and Arthroscopy; SD, standard deviation.

CIA, on the other hand, showed greater variability and was supported by smaller sample sizes across studies. As shown in Figure 5, values largely overlapped across techniques and clustered within the native ACL range and below the ESSKA consensus threshold, and the clinical relevance of reproducing native coronal inclination remains uncertain.

Over the past three decades, ACL reconstruction has evolved through multiple technical iterations; however, femoral tunnel malposition remains a leading cause of graft failure and suboptimal outcomes [5, 8, 19]. MRI‐based assessment of graft orientation offers an objective and reproducible means of evaluating reconstruction strategies. The OTT technique eliminates the need for femoral tunnel creation, thereby avoiding a major source of technical variability [25]. In addition, preservation of the tibial graft attachment maintains vascularity [53], which has been associated with improved graft integration in experimental [29, 38] and clinical studies and with superior MRI‐based integration compared with devascularized grafts [14, 28, 43]. However, the absence of a femoral tunnel does not eliminate all potential sources of graft malalignment or instability, none of which were evaluated in this imaging‐based study.

**Figure 6:**
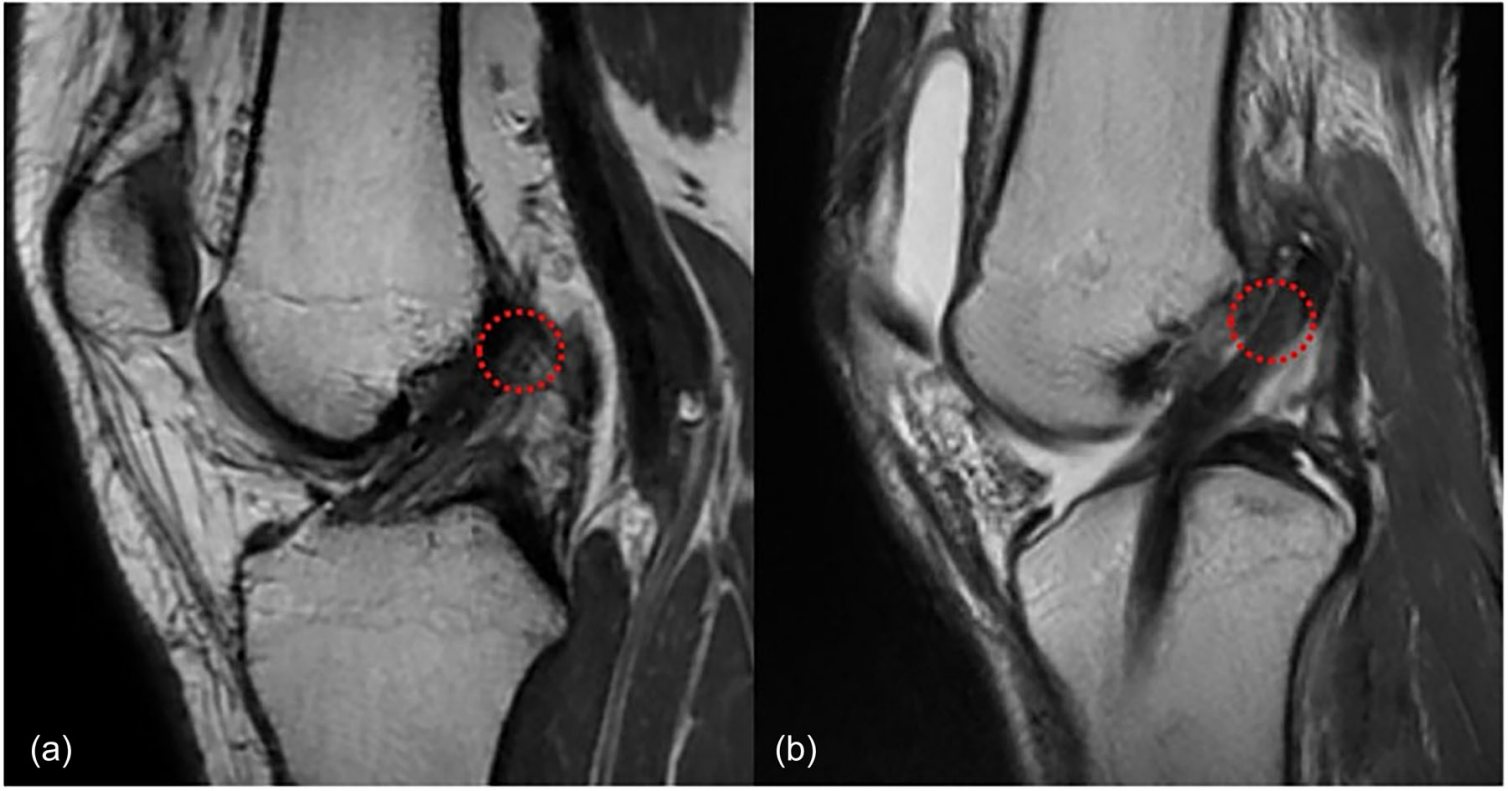
(a, b) Illustration of how the over‐the‐top technique approximates the native femoral attachment of the ACL, highlighted by the dotted red circle. ACL, anterior cruciate ligament.

A key strength of this study is that it represents the first objective assessment of the anatomicity of OTT ACL reconstruction using MRI‐based inclination measures, overcoming the limitations of tunnel‐dependent evaluation methods and allowing meaningful comparison with other reconstruction strategies (Figure 6).

Several limitations should be acknowledged. The contralateral limb was not imaged in the surgical cohort; therefore, an independent control group was used to provide native ACL reference values. The groups differed in age and sex distribution, and no adjusted analysis was performed; thus, comparisons should be interpreted as descriptive rather than isolated technique effects. This study evaluated graft orientation using SIA and CIA as surrogate imaging markers and did not assess full three‐dimensional anatomy or in vivo biomechanical behaviour. Clinical outcomes were not analysed in the present investigation, as the study was designed specifically as an imaging‐based assessment; however, clinical results following OTT ACL reconstruction have been reported previously by our group and others [15, 33, 54]. Finally, variability in MRI acquisition and positioning may have influenced measurements, although interobserver reliability was excellent.

## CONCLUSION

OTT ACL reconstruction produces graft inclination angles that are significantly greater than those of the native ACL but frequently fall within consensus‐recommended MRI thresholds for acceptable alignment. MRI‐based inclination measures provide objective structural characterisation of a technique historically described as ‘non‐anatomic’. These imaging findings should be interpreted alongside previously reported long‐term clinical outcomes for OTT reconstruction, as inclination angles alone do not define anatomic restoration or functional equivalence. Rather, the present study complements existing outcome literature by providing reproducible structural data within a standardised MRI framework.

## AUTHOR CONTRIBUTIONS

Mok Ying Ren performed the statistical analysis and drafted the manuscript. Andrea Pierangeli, Lidia Ana Martín Domínguez and Claudio Rossi independently reviewed imaging data and compiled the dataset. Alberto Grassi, Giulio Maria Marcheggiani Muccioli and Stefano Zaffagnini were the lead surgeons responsible for the surgical procedures included in this study. All authors critically reviewed the manuscript and approved the final version.

## CONFLICTS OF INTEREST STATEMENT

Alberto Grassi, MD: Smith & Nephew: Not paid consultant. Stefano Zaffagnini, MD, Professor: DePuy, a Johnson & Johnson Company: Paid presenter or speaker; paid consultant and European Society of Sports Traumatology, Knee Surgery and Arthroscopy (ESSKA): Board or committee member, International Society of Arthroscopy, Knee Surgery, and Orthopaedic Sports Medicine (ISAKOS): Board or committee member, Journal of Experimental Orthopaedics (JEO): Editorial or governing board and Smith & Nephew: Paid presenter or speaker; paid consultant.

## ETHICS STATEMENT

Please include the name of the institutional review board (IRB) and the approval number. If not applicable, please state so. The study protocol was approved by the Area Vasta Emilia Centro Ethics Committee (PG 0000445)—see attached.

## Supporting information

Supporting File

## Data Availability

The data that support the findings of this study are available from the corresponding author upon reasonable request.
