# Supplementary material for: Graft orientation using the over‐the‐top technique in anterior cruciate ligament reconstruction: Magnetic resonance imaging‐based study
Source: J Exp Orthop. 2026 May 6;13(2):e70744. doi: 10.1002/jeo2.70744 (PMC13147152; doi:10.1002/jeo2.70744)
Supplement: Supplementary file 1 — Supporting File [file JEO2-13-e70744-s001.docx]

**Supplementary Table S1: Studies contributing to reference values**

| **Study** | **N** | **Age** | **Gender** | **SIA (°)** | **CIA (°)** |
| --- | --- | --- | --- | --- | --- |
| **Native ACL** | | | | | |
| Ayerza et al 2003 | 30 | 30.1 ± 8.6 | NA | 51.4 ± 3.2 |  |
| Mellado et al 2004 | 50 | 41.5 ± 13.1 | 31M/ 19F | 53.5 ± 3.7 |  |
| Fujimoto et al 2004 | 10 | NA | NA |  | 67.0 ± 4.2 |
| Ahn et al 2007 | 50 | 28.3 ± 9.9 | 39M/ 11F | 58.7 ± 3.8 | 65.9 ± 4.4 |
| Andrei et al 2015 | 74 | NA | NA | 51.9 ± 1.95 |  |
| Illingworth et al 2011 | 50 | NA | NA | 49.9 ± 2.8 |  |
| Cho et al 2012 | 15 | NA | NA | 50.8 ± 2.1 |  |
| Cho et al 2012 | 15 | NA | NA | 50.9 ± 2.4 |  |
| Guler et al 2016 | 25 | 31.4 ± 4.1 | 25M / 0F | 46.23 ±4.86 | 62.49 ±3.79 |
| Guler et al 2016 | 23 | 30.9 ± 3.7 | 22M/ 1F | 46.08 ±2.28 | 62.12 ±2.07 |
| Reid et al 2017 | 188 | 152 Mature/ 36 Immature | 98M/ 90F | 46.9 ± 4.9 | 74.3 ± 4.7 |
| Stone et al 2019 | 15 | 23.79 ± 3.29 | 11M 4F | 51.86 ±2.96 |  |
| Stone et al 2019 | 14 | 24.26 ± 3.62 | 11M 3F | 50.83 ±5.83 |  |
| Jamsher et al 2024 | 18 | 38.0 ± 11.4 | 13M/ 5F | 49.3 ± 4.2 | 73.6 ± 3.4 |
| Present Study | 28 | 33.1 ± 12.8 | 17M/ 11F | 49.6 ± 5.6 | 63.1 ± 5.5 |
| **Outside-in/ Retrograde Drilling** | | | | | |
| Ahn et al 2017 | 39 | 31.4 ± 11.1 | 33M/ 6F | 56.04 ±4.57 | 70.60 ±7.51 |
| Jamsher et al 2024 | 18 | 29.4 ± 8.3 | 14M/ 4F | 50.5 ± 6 | 69.0 ± 7.2 |
| Yang et al 2024 | 122 | 28.7 ± 12 | 100M/ 22F | 51.5 ± 5.4 |  |
| Yang et al 2024 | 54 | 26.7 ± 10.6 | 44M / 10F | 51.8 ± 5.0 |  |
| **Anteromedial Portal Drilling (Flexible)** | | | | | |
| Lee et al 2023 | 60 | 28.4 ± 9.9 | 37M/ 23F | 52.4 ± 4.6 | 69.2 ± 4.7 |
| Jamsher et al 2024 | 18 | 33.4 ± 12.6 | 13M/ 5F | 49.9 ± 5.0 | 69.3 ± 4.5 |
| **Over The Top** | | | | | |
| Present Study | 35 | 26.1 ± 8.1 | 29M/ 6F | 53.2 ± 5.1 | 71.3° ± 5.9 |
| **Anteromedial Portal Drilling (Rigid)** | | | | | |
| Ayerza et al 2003 | 30 | 32.3 ± 8.7 | NA | 66.7 ± 5.9 |  |
| Illingworth et al 2011 | 16 | NA | NA | 51.8 ± 6.5 |  |
| Cho et al 2012 | 15 | NA | NA | 51.6 ± 3.3 |  |
| Andrei et al 2015 | 74 | NA | NA | 52.6 ± 2.9 |  |
| Guler et al 2016 | 23 | 30.9 ± 3.7 | 22M/ 1F | 53.22 ±1.73 | 62.37 ±1.94 |
| Stone et al 2019 | 14 | 24.26 ± 3.62 | 11M 3F | 52.57 ±4.75 | 48.06 ±4.74 |
| Jamsher et al 2024 | 18 | 27.5 ± 7.2 | 17M/ 1F | 56.0 ± 6.1 | 69.5 ± 5.3 |
| **Transtibial Drilling** | | | | | |
| Fujimoto et al 2004 | 30 | 26.3 (15 – 53) | 18M/ 12F |  | 74.9 ± 7.5 |
| Ahn et al 2007 | 50 | 31.7 ± 9.9 | 40M/ 10F | 64.6 ± 4.2 | 73.5 ± 2.4 |
| Ahn et al 2007 | 46 | 29.4 ± 8.4 | 35M/ 11F | 71.3 ± 6.0 | 75.2 ± 2.9 |
| Illingworth et al 2011 | 34 | NA | NA | 63.5 ± 7.0 |  |
| Cho et al 2012 | 15 | NA | NA | 59.9 ± 5.7 |  |
| Guler et al 2016 | 25 | 31.4 ± 4.1 | 25M / 0F | 58.21 ±4.85 | 70.51 ±1.87 |
| Rose et al 2017 | 16 | 45.0 ± 12.5 | 7M/ 9F | 54.6 ± 6.4 | 64.9 ±4.2 |
| Rose et al 2017 | 16 | 37.0 ± 12.0 | 11M/ 5F | 56.5 ± 8.3 | 66.8 ± 7.3 |
| Ahn et al 2017 | 42 | 29.7 ± 9.36 | 35M/ 7F | 61.0 ± 7.08 | 76.73 ±5.28 |
| Stone et al 2019 | 15 | 23.79 ± 3.29 | 11M 4F | 52.41 ±4.72 | 57.28 ±9.29 |
| Jamsher et al 2024 | 18 | 32.9 ± 9.3 | 14M/ 4F | 58.9 ± 5.3 | 73.1 ± 5.3 |

**Supplementary Table S1.** Characteristics of MRI-based studies reporting sagittal and coronal ACL inclination angles. Included studies employed validated measurement definitions consistent with ESSKA Consensus, using the tibial long axis as an objective reference. Studies utilizing non-standard or arbitrary sagittal inclination measurements without reference to the tibial long axis were excluded.
